# Deciphering the Dynamics of Signaling Cascades and Virulence Factors of *B. cinerea* during Tomato Cell Wall Degradation

**DOI:** 10.3390/microorganisms9091837

**Published:** 2021-08-30

**Authors:** Almudena Escobar-Niño, Inés M. Morano Bermejo, Rafael Carrasco Reinado, Francisco Javier Fernandez-Acero

**Affiliations:** Microbiology Laboratory, Institute for Viticulture and Agri-Food Research (IVAGRO), Marine and Environmental Sciences Faculty, University of Cádiz, 11510 Puerto Real, Spain; almudena.escobar@uca.es (A.E.-N.); inesm.morano@gmail.com (I.M.M.B.); rafael.carrasco@uca.es (R.C.R.)

**Keywords:** *Botrytis cinerea*, proteome, signaling, pathogenic factors, virulence factors, toxins, tomato

## Abstract

The ascomycete *Botrytis cinerea* is one of the most relevant plant pathogenic fungi, affecting fruits, flowers, and greenhouse-grown crops. The infection strategy used by the fungus comprises a magnificent set of tools to penetrate and overcome plant defenses. In this context, the plant-pathogen communication through membrane receptors and signal transduction cascades is essential to trigger specific routes and the final success of the infection. In previous reports, proteomics approaches to *B. cinerea* signal transduction cascades changes in response to different carbon source and plant-based elicitors have been performed. Analyzing the secretome, membranome, phosphoproteome, and the phosphomembranome. Moreover, phenotypic changes in fungal biology was analyzed, specifically toxin production. To obtain the whole picture of the process and reveal the network from a system biology approach, this proteomic information has been merged with the phenotypic characterization, to be analyzed using several bioinformatics algorithms (GO, STRING, MCODE) in order to unravel key points in the signal transduction regulation crucial to overcome plant defenses, as well as new virulence/pathogenicity factors that could be used as therapeutic targets in the control of the gray mold rot disease. A total of 1721 and 663 exclusive or overexpressed proteins were identified under glucose (GLU) and deproteinized tomato cell walls (TCW), summarizing all of the protein identifications under phenotypic characterized stages. Under GO analysis, there are more biological process and molecular functions described in GLU, highlighting the increase in signaling related categories. These results agree with the high number of total identified proteins in GLU, probably indicating a more varied and active metabolism of the fungus. When analyzing only GO annotations related with signal transduction, it was revealed that there were proteins related to TOR signaling, the phosphorelay signal transduction system, and inositol lipid-mediated signaling, only under GLU conditions. On the contrary, calcium-mediated signaling GO annotation is only present between the proteins identified under TCW conditions. To establish a potential relationship between expressed proteins, cluster analyses showed 41 and 14 clusters under GLU and TCW conditions, confirming an increase in biological activity in GLU, where we identified a larger number of clusters related to transcription, translation, and cell division, between others. From these analyses, clusters related to signal transduction and clusters related to mycotoxin production were found, which correlated with the phenotypic characterization. The identification of the proteins encompassed in each condition and signal transduction cascade would provide the research community with new information about the *B. cinerea* infection process and potential candidates of pathogenicity/virulence factors, overcoming plant defenses, and new therapeutic targets.

## 1. Introduction

In regard to plant diseases, gray mold, caused by the phytopathogenic fungi *Botrytis cinerea,* is one of the most devastating. This disease attacks hundreds of agronomic crops around the world, especially tomatoes and grapes. There has been more research conducted on crop maintenance and the development of new botricides in response to the economic losses that farmers have faced [1].

Since the *B. cinerea* genome was first published, and the most recent genomic assembly performed [2,3], molecular approaches to *B. cinerea* have been facilitated, particularly innovative “omics” technologies, such as transcriptomics and proteomics. Most of this information has been used to determine the role as virulence or pathogenicity factors of several genes. The “pathogen–host interaction database” [4] (http://www.phi-base.org/, accessed on 20 May 2021) lists 391 different genes from *B. cinerea*, most of them related to infective machinery. However, even the collected molecular information is increasing; there is no fungicide based on this biological information [5]. 

Most of the detected genes with crucial roles in the infective processes are genes related to the signaling cascades. These proteins are a crucial bridge between environmental changes outside the cell to the activation or inhibition of specific enzymatic routes, to produce plant invasion and fungal growth. To collect as much information as possible on the proteins involved in the *B. cinerea* signaling machinery, several proteomics approaches were developed. At the end of the signaling process from outside the cell to the nucleus, the secretome of *B. cinerea* was revealed, showing specific fungal responses to different plant based elicitors [6]. To collect the next level of signaling processes, membrane proteins were collected and analyzed [7]. Finally, the phosphoproteome [8] and the phosphomembranome [9] were studied under the same culture conditions. All of these approaches have detected several proteins with potential roles as virulence/pathogenicity factors, but the fungal and environmental global relationship. Moreover, the molecular virulence triggers remain un-dilucidated. 

These proteomics approaches were developed using two different carbon sources, glucose as a constitutive phase and deproteinized cell walls from tomatoes to induce fungal virulence. The relation between the obtained proteins and the used carbon sources was determined [6]. Moreover, the phenotypic characterization, during the supposed constitutive state with glucose, showed that the amount of toxins (botryoidal and dihydrobotrydial) was maximum, whereas this production disappeared under virulence induction using TCW as a sole carbon source. Moreover, while cell wall degrading enzyme (CWDE) was massive with TCW, it presents minimum levels under glucose induction [10]. The relation between both processes suggest that there is a coordinated control. Initially, *B. cinerea* detected the plant cell walls and produced CWDE, to produce plant invasion; then when glucose was obtained from plant macerated tissues, the fungus produced a toxin to suppress cellular plant defenses and kill the cells. To obtain a complete map of this interaction, all of this information was merged and analyzed using several bioinformatics algorithms (GO, STRING, MCODE) in order to unravel key points in the signal transduction regulation, crucial to overcome plant defenses, as well as new virulence/pathogenicity factors that could be used as therapeutical targets in the control of gray mold rot disease.

## 2. Material and Methods

### 2.1. Fungal Strains and Culture Conditions

*B. cinerea* B05.10 (provided by Dr. Paul Tudzunski from the University of Münster, Münster, Germany) was the strain used in this study. Conidial stock suspensions were prepared and maintained as previously reported [8]. Two different carbon sources were used: glucose (GLU) (Panreac, Spain) as the constitutive stage; and deproteinized tomato cell walls (TCW) as the virulence inductor, as previously described by Fernandez-Aceroet et al. (2010) [6]. In sum, 500 mL flasks containing 250 mL of minimal salt medium (MSM) (50 mM NH_4_Cl, 7.3 mM KH_2_PO_4_, 4.2 mM MgSO_4_, 6.7 mM KCl, 0.07 mM FeSO_4_) supplemented with 1% carbon source assayed, were inoculated with *B. cinerea* conidia, to a final concentration of 5 × 10^4^ conidia/mL. Four independent replicas were assayed per culture condition. Replicates were incubated in parallel at 180 rpm at 22 °C under alternating 12-h light/dark cycles for 5 days. After 5 days, PhosSTOP Phosphatase Inhibitor Cocktail (Roche, Basilea, Switzerland) was added to the culture according to the manufacturer’s instructions. Then, mycelia and culture medium were separated by filtration in a 30-µm nylon filter (Sefar Nytal, Heiden, Switzerland) and stored at −80 °C until use for protein extraction.

### 2.2. Isolation of Proteins 

For secretome isolation, the culture media were sequentially centrifuged for 20 min at 5000× *g*, and 30 min at 15,000× *g*. The obtained media were used for subsequent protein precipitation using a deoxycholate (DOC)/trichloroacetic acid (TCA) method, as previously described [11]. The digestion of proteins and sample preparation for MS analysis was performed as described by Fernandez-Acero et al. (2010) [6]. *B. cinerea* membranome was obtained from ground mycelium using ReadyPrep Protein Extraction Kit (membrane I) (Bio Rad, Hercules, CA, USA) and acetone precipitation, as previously described byLineiro et al. (2016) [7]. Protein digestion and sample preparation for MS analysis was performed as previously described [12]. Protein extraction in phosphoproteome was carried out, treating powdered mycelium with a phenol-based procedure, as previously reported [6]. The protein extract was through phosphopeptide enrichment using titanium dioxide and MS sample preparation, as described byLineiro et al. (2016) [12]. Finally, phosphomembranome was isolated by the temperature-dependent partition method [13] with minor modifications [7] using powdered mycelium. The membrane extract was enriched in phosphorylated membrane proteins and prepared to MS analysis as described by Escobar-Nino et al. (2019) [9].

### 2.3. Proteomes Data Integration

*Botrytis cinerea* subproteomes identification were performed as previously reported by our group [6,9,12]. These analyses included *B. cinerea* secretome, membranome, phosphoproteome, and phosphomembranome. All of these subproteomes were obtained after 5 days of growth under two different culture conditions, GLU and TCW. The datasets generated during these studies are available in the PRIDE repository, (https://www.ebi.ac.uk/pride/archive/, accessed on 20 May 2021) with the dataset identifiers PXD003099 (membranome); PXD003099 (phosphoproteome); and PXD010961 (phosphomembranome). Exclusive or overexpressed proteins identified under GLU or TCW conditions in these subproteomes were integrated in a sole table for each carbon source, eliminating duplicated accession numbers (Supplementary Table S1A,B). The remaining proteins identified under each culture condition were analyzed as a whole proteome.

### 2.4. Bioinformatic Analysis

GO annotation was performed using the following procedure. All 2384 identified proteins were re-annotated by OmicsBox (v.1.3.11) to actualize all associated functional information. The nr database (date 27 March 2020) was employed as a Blastp query searching for homologies; and the public EMBL-EBI InterPro web service was used to scan sequences against InterPro signatures. Furthermore, GO-enzyme code mapping was employed to map annotated Gene Ontology terms to enzyme codes, allowing to retrieve metabolic pathways based on the associated GO terms and enzyme codes. Finally, to identify the functional groups significantly overrepresented in the two compared samples, a Single Enrichment Analysis (SEA), using a two-tailed Fisher’s Exact test, was employed to contrast GO annotations associated to proteins more abundant in glucose (reference) or a tomato cell wall (test) using a threshold FDR < 0.01. Two kinds of SEA analysis were performed: (i) one with all levels of GO annotation; and (ii) a second one using only highest levels of GO annotation (reduced SEA). In addition, the STRING protein interaction database (v.11.0) (https://string-db.org/, accessed on 20 May 2021) [14] was used to generate a protein interaction network of all 2384 identified proteins (medium confidence _ 0.4). The protein–protein network obtained was then imported into Cytoscape (version 3.8.0) [15] and the clustering algorithm MCODE (version 1.5.1) [16] was run to identify potential functional clusters (Degree cutoff = 2; Haircut; Node score cutoff = 0.2; K-Core = 2; Max. Depth = 100). Predicted clusters were classified according to the functioning of the proteins that made them up. Finally, proteins identified in GO analysis and cluster identification related to signal transduction were studied deeper, using Blast [17] and NCBI Conserved Domain Search tools [18], especially those without clear annotations assigned in the *B. cinerea* genome databases (hypothetical proteins).

## 3. Results

### 3.1. Gene Ontology Classification

**Figure 1:**
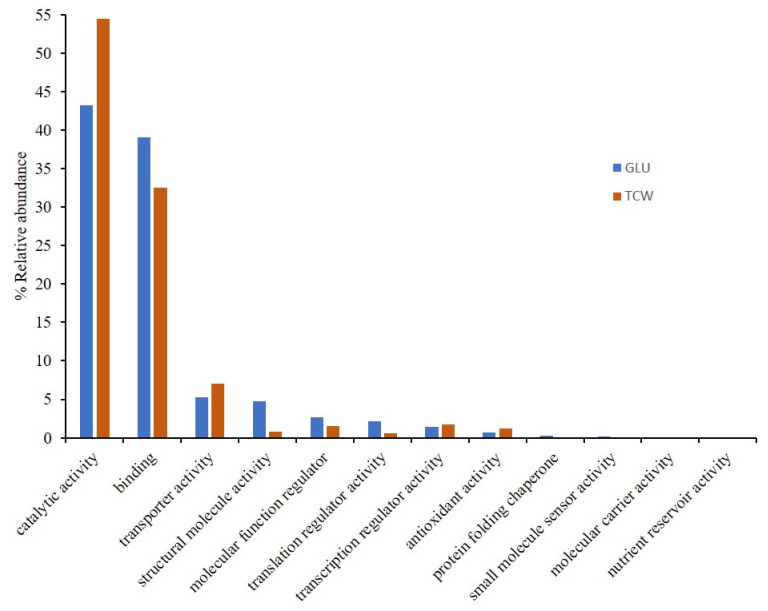
Gene ontology (GO) classification by the molecular function (level 2 of categorization) of proteins exclusively identified or overexpressed in *B. cinerea* proteomes (membranome, phosphoproteome, phosphomembranome, and secretome), under GLU and TCW conditions, as the sole carbon source. Relative abundance represents the percentage of membrane-associated phosphoproteins identified in each category relative to the total number of protein GO annotations in this level.

**Figure 2:**
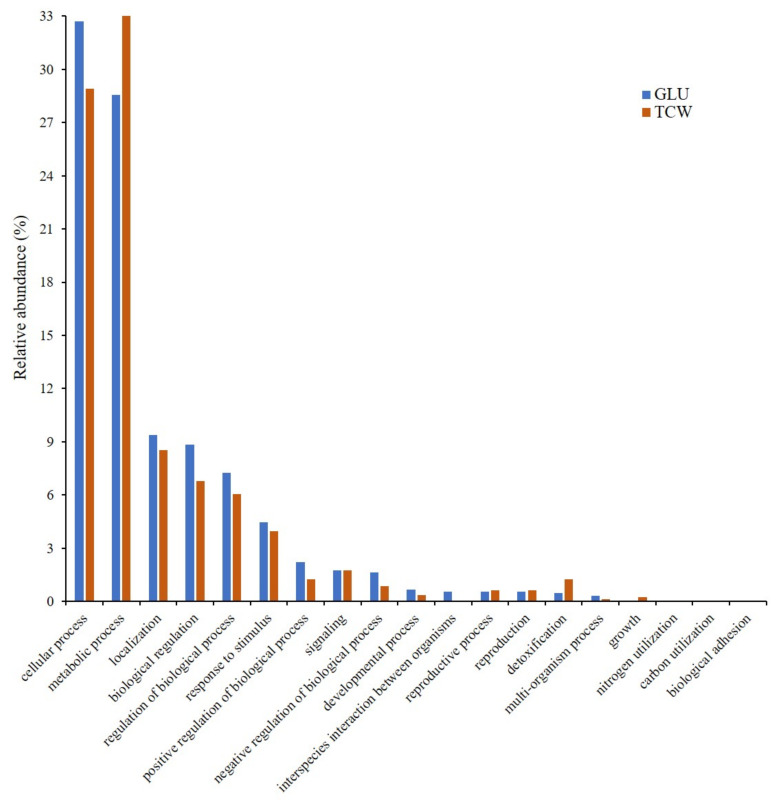
Gene ontology (GO) classification by the biological process (level 2 of categorization) of proteins, exclusively identified or overexpressed in *B. cinerea* proteomes (membranome, phosphoproteome, phosphomembranome, and secretome), under GLU and TCW conditions, as the sole carbon source. Relative abundance represents the percentage of membrane-associated phosphoproteins identified in each category relative to the total number of protein GO annotations in this level.

To obtain a complete view of the process, *B. cinerea* proteomes information, previously obtained [6,9,12], was unified. A total of 1721 and 663 exclusive or overexpressed proteins were identified under each used carbon source, glucose (GLU) and deproteinized tomato cell walls (TCW) (Supplementary Table S1). Both sets of proteins were used in several bioinformatic analysis, from gene ontology (GO) analysis to protein interaction studies. GO analysis was performed in order to determine the biological relevance of *B. cinerea* proteins identified in the different proteomes performed by our groups, and that were affected by the changes in the used carbon source. With this main aim, exclusive or overexpressed proteins identified under each condition were categorized according to their specific gene ontology (GO) annotations, by molecular functions (MF) (Figure 1) and biological processes (BP) (Figure 2).

Classification by MF showed a higher number of categories represented in the GLU condition than in TCW. In fact, there are four categories without any proteins representing them when TCW is present as the sole carbon source: (i) protein folding chaperone; (ii) small molecule sensor activity; (iii) molecular carrier activity; and (iv) nutrient reservoir activity (Figure 1). In the same way, BP classification (Figure 2) showed similar results, with a higher number of categories represented under the GLU condition than under TCW. As in the molecular function, there were four categories without any annotated proteins when TCW was present as the sole carbon source: (i) interspecies interaction between organisms; (ii) nitrogen utilization; (iii) carbon utilization; and (iv) biological adhesion. 

SEA analysis of BP and MF showed the increasing of: (i) the energy consumption processes, such as production of new proteins, DNA- and RNA-binding, and ATP-binding in GLU; and (ii) cell wall degradation and glucose import proteins in TCW (Supplementary Table S2; and Supplementary Figures S1 and S2).

**Figure 3:**
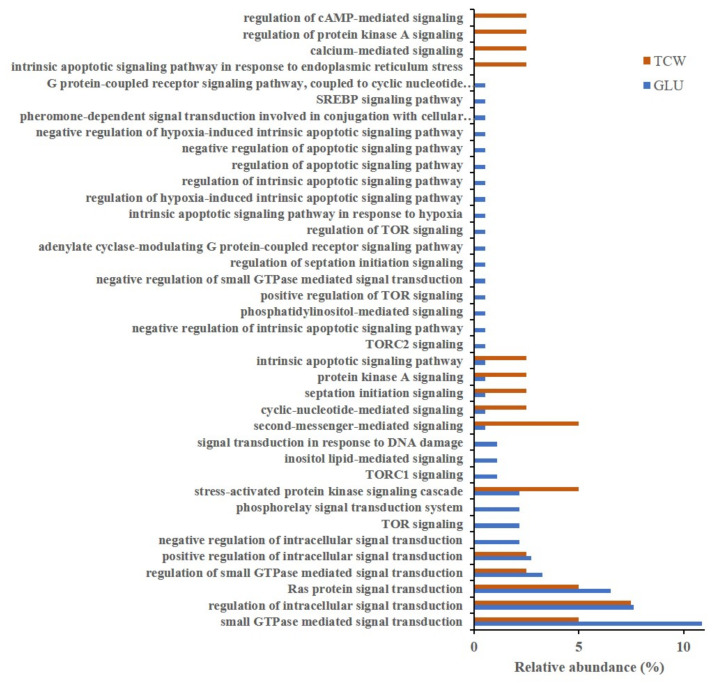
Gene ontology (GO) classification by the biological process of categories related to signaling (level 5 and 6)) of proteins exclusively identified or overexpressed in *B. cinerea* proteomes (membranome, phosphoproteome, phosphomembranome, and secretome), under GLU and TCW conditions, as the sole carbon source. Relative abundance represents the percentage of proteins identified in each category relative to the total number of protein GO annotations in this level.

As the main aim of this approach was to settle the analysis of plant–pathogen communication, the BP category related to signaling, presenting the same percentage of relative abundance in both conditions (Figure 2), was dissected, and higher levels of GO annotation were analyzed and represented (Figure 3). This approach showed an increase in signaling-related categories under the GLU condition, specifically proteins related to TOR signaling, the phosphorelay signal transduction system, and inositol lipid-mediated signaling, only under GLU conditions. 

Focusing on the GLU condition, the Tor signaling category is mainly represented by an identified putative phosphatidylinositol 3-kinase TOR protein (hypothetical protein BCIN_01g11360). Target of rapamycin (TOR) proteins are serine/threonine phosphoinositide kinases that acts as master regulators to control cell growth by integrating nutrients, energy, and growth factors in eukaryotes [19]. Additionally, one putative regulator and one putative effector of the TOR complex were identified in this category: (i) hypothetical protein BCIN_02g06630; and (ii) Bcsec13. An NCBI Conserved Domain Search showed that the hypothetical protein BCIN_02g06630 includes a PH_Slm1: Slm1 Pleckstrin homology (PH) domain (cd13311).

The phosphorelay signal transduction category involves the transfer of phosphate groups between histidine and aspartate, which then act as the phospho-donor to response regulator proteins. This category, under the GLU condition, was represented by Bchhk2 (HHK2, histidine kinase-group V protein); Bcrim15 (serine/threonine protein kinase RIM15); and Bcos1 (histidine kinase).

The last of the most relevant categories exclusively presented under the GLU condition is the inositol lipid-mediated signaling category, which includes phosphatidylinositol-mediated signaling. Those proteins involve a series of signals in which a cell uses an inositol-containing lipid to convert a signal into response. This category contains two proteins: (i) Bcstt4 (phosphatidylinositol 4-kinase STT4); and (ii) a putative phospholipase D1 (hypothetical protein BCIN_09g01240). A Blastp analysis of hypothetical protein BCIN_09g01240 showed high similarity of this protein with the putative phospholipase d1 protein of *Botrytis cinerea* BcDW1 (EMR83559.1; 99.94% of identity and 100% of cover) and with a protein similar to phospholipase D1 (PLD1) of *Botrytis cinerea* T4 (CCD55075.1; 98.34% of identity and 100% of cover). NCBI’s Conserved Domain Search tool revealed, as the best hits, domains of phospholipase D (PLD) proteins (PLN02866, cd09141), suggesting a potential PLD function of this hypothetical protein.

Another nine GO categories were exclusively represented under the GLU conditions: (1) negative regulation of intrinsic apoptotic signaling pathway; (2) intrinsic apoptotic signaling pathway in response to hypoxia; (3) regulation of hypoxia-induced intrinsic apoptotic signaling pathway; (4) regulation of intrinsic apoptotic signaling pathway; (5) regulation of apoptotic signaling pathway; (6) negative regulation of apoptotic signaling pathway; (7) negative regulation of hypoxia-induced intrinsic apoptotic signaling pathway; (8) adenylate cyclase-modulating G protein-coupled receptor signaling pathway; (9) and G protein-coupled receptor signaling pathway, coupled to the cyclic nucleotide second messenger. One to seven categories are related to the regulation of apoptotic signaling and they are only represented by the protein Bclhs1; categories 8 and 9 belong to the adenylate cyclase-modulating GPCR signaling pathway, and they are only represented by Bcg2. 

On the other hand, there were four GO categories present only under TCW as the sole carbon source: (1) intrinsic apoptotic signaling pathway in response to endoplasmic reticulum stress; (2) calcium-mediated signaling; (3) regulation of protein kinase A signaling; and (4) regulation of cAMP-mediated signaling. Calcium-mediated signaling is represented by a protein identified in the membranome of *B. cinerea* [7], the Calmodulin (Bc4). On the other hand, regulation of protein kinase A signaling and regulation of cAMP-mediated signaling GO categories under TCW is represented by the cAMP-dependent protein kinase regulatory subunit (BcPKAR). The last category exclusively identified under TCW was the intrinsic apoptotic signaling pathway in response to endoplasmic reticulum stress. This category contained one protein, a homologous of yeast Ire1p (Bcire1).

### 3.2. Protein Interaction Analysis

In order to unravel the signal transduction cascades differentially activated under each carbon source, we performed a protein interaction analysis using the STRING database and Cytoscape [15,20]. Exclusive or overexpressed proteins identified under each condition of *B. cinerea* proteomes were used (Supplementary Table S1), in order to unravel new protein–protein interactions that could not be identified in the individual analysis of each proteome. Firstly, we searched our identified proteins against *B. cinerea* proteins in the STRING database, which is a database of known and predicted protein–protein interactions. The interactions included direct (physical) and indirect (functional) associations, stemming from computational prediction, from knowledge transfers between organisms, and from interactions obtained from other databases. By using the identified proteins in each condition, the analysis returned a network of 262 nodes (proteins) and 504 connecting edges (predicted functional associations) in TCW (Supplementary Table S3) and a network of 1295 interacting nodes and 15,483 connecting edges in GLU (Supplementary Table S4). These networks were through a deeper analysis by using the software MCODE [16]. MCODE finds clusters (highly interconnected regions) in a network. Clusters in a protein–protein interaction network are often protein complexes and parts of pathways. This analysis returned 14 and 41 clusters identified in TCW and GLU, respectively (Supplementary Tables S5 and S6). Under the GLU condition, 3 out of the 41 clusters were related to signal transduction and one was related to toxin production. In contrast to that, only one cluster related to signaling was identified under the TCW condition. 

#### 3.2.1. Cluster Analysis under the GLU Condition

**Figure 4:**
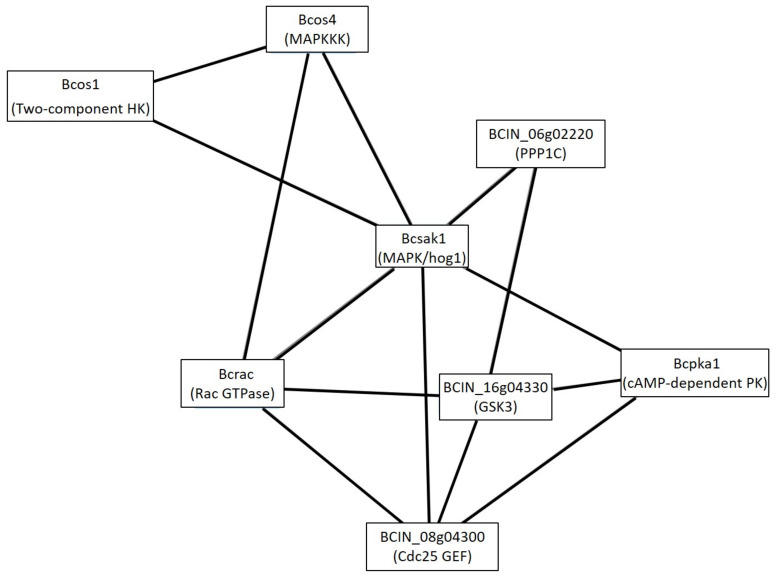
Cluster 15: signaling cluster under the GLU condition. Clusters were obtained analyzing STRING networks of protein exclusively identified or overexpressed in *B. cinerea* proteomes (membranome, phosphoproteome, phosphomembranome, and secretome) under GLU with MCODE.

When glucose was present as a sole carbon source, three clusters relating to signal transduction were detected. The first was Cluster 15, which had more well-known signaling proteins (Figure 4). The signal transduction cascades implicated in this cluster were small GTPases (Bcrac, BCIN_08g04300), cAMP Cascade (Bcpka1), MAP kinases Cascades (Bcsak1, BcOs4) and the two-component signal transduction system (Bcos1). In addition, the cluster predicted some direct or indirect interaction between its members, such as the interaction of Bcsak1 and cAMP-dependent kinase Bcpka1; interaction of the histidine kinase (HK) Bcos1 and MAPK pathway (bcsak1 and Bcos4); interaction between Bcrac and the MAP kinases (Bcsak1); and a link between Bcrac and BCIN_08g04300, which is connected to Bcpka1. An NCBI Conserved Domain Search (CD-search) showed that the hypothetical protein BCIN_08g04300 sequence contained domains SH3_Sdc25; WW; REM; and RasGEF. This protein belongs to the cd11883: SH3_Sdc25 subfamily, which is composed of the *Saccharomyces cerevisiae* guanine nucleotide exchange factors (GEFs) Sdc25 and Cdc25, and similar proteins. The last members of this cluster were glycogen synthase kinase 3 (GSK3: BCIN_16g04330) and a putative serine/threonine protein phosphatase PP1C (BCIN_06g02220).

**Figure 5:**
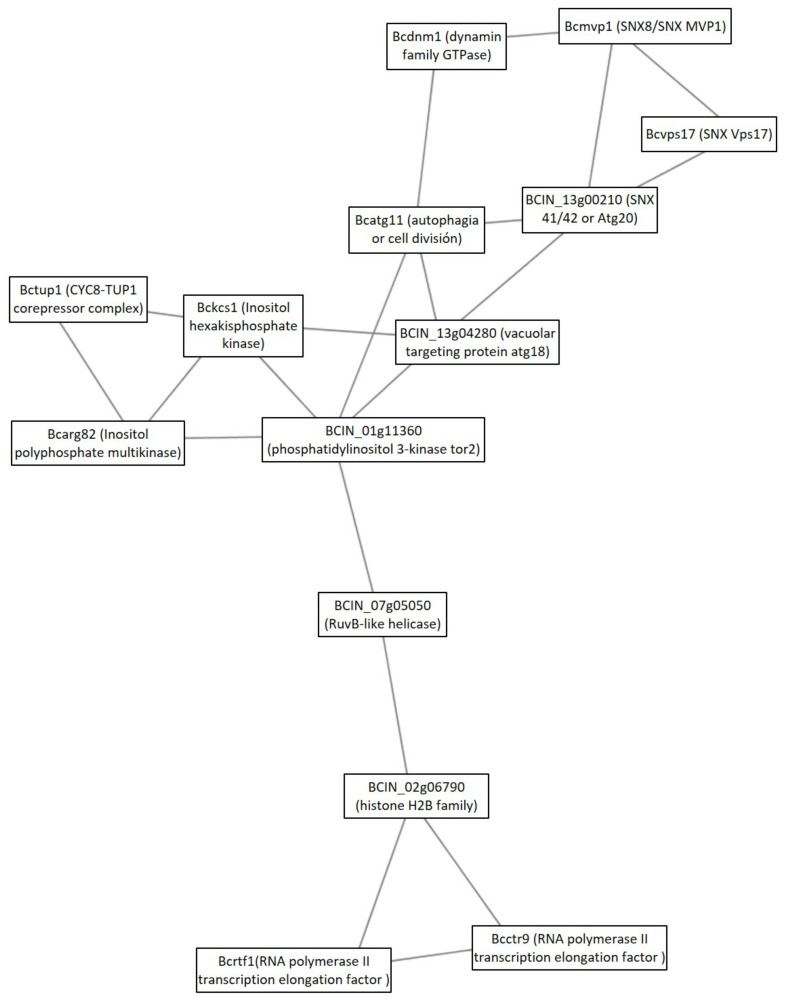
Cluster 24: signaling cluster under the GLU condition. Clusters were obtained analyzing STRING networks of protein, exclusively identified or overexpressed in *B. cinerea* proteomes (membranome, phosphoproteome, phosphomembranome, and secretome) under GLU with MCODE.

The second signaling cluster was Cluster 24. The main component of this cluster is the aforementioned component of TOR signaling, a putative phosphatidylinositol 3-kinase tor2 (BCIN_01g11360), which seems to connect and regulate three groups of proteins (Figure 5). The first group of proteins of the cluster is composed of proteins that participate in membrane trafficking processes along the endolysosomal pathway (Bcdnm1, Bcmvp1, BCIN_13g00210, Bcatg11, BCIN_13g04280). Three of them are potential sorting nexins (SNXs), such as Bcmvp1 (SNX8/SNX Mvp1), Bcvps17 (SNX Vps17), and BCIN_13g00210 (SNX 41/42). The second group of proteins connected to phosphatidylinositol 3-kinase tor2 is composed of proteins related to phosphatidylinositol signaling system and metabolism (Bckcs1, Bcarg82, BCIN_01g11360). The last group of proteins of cluster 24 was hypothesized to be related to transcription (BCIN_07g05050, BCIN_02g06790, Bcrtf1, and Bcctr9). Blastp and a Conserved Domain Search analysis showed that BCIN_07g05050 was a putative RuvB-like helicase; and BCIN_02g06790 was a histone H2B family protein. 

**Figure 6:**
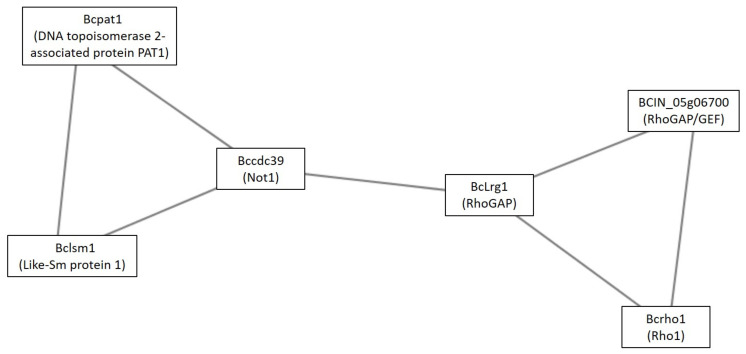
Cluster 36: signaling cluster under GLU condition. Clusters were obtained analyzing STRING networks of protein exclusively identified or overexpressed in *B. cinerea* proteomes (membranome, phosphoproteome, phosphomembranome, and secretome) under GLU with MCODE.

Cluster 36 was the last signaling cluster identified under GLU condition (Figure 6). This was composed of small GTPase cascade components (Bclrg1, Bcrho1, and BCIN_05g06700) and mRNA decay members (Bcpat1, Bclsm1, and Bccdc39). A Conserved Domain Search using the BCIN_05g06700 protein sequence showed domains of Rho1GAP (specific hit: RhoGAP_fSAC7_BAG7/cd04396) and RhoGEF (non-specific hit: ROM1/COG5422).

**Figure 7:**
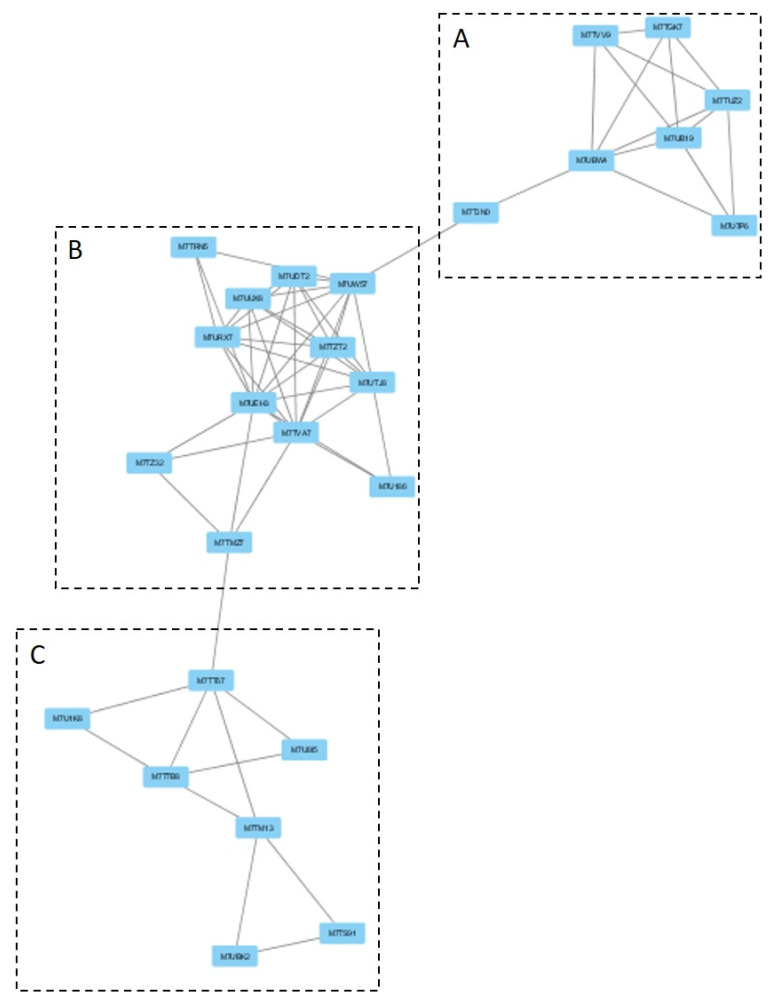
Cluster 10: toxin production cluster under the GLU condition. Clusters were obtained analyzing STRING networks of protein, exclusively identified or overexpressed in *B. cinerea* proteomes (membranome, phosphoproteome, phosphomembranome, and secretome) under GLU with MCODE.

**Figure 8:**
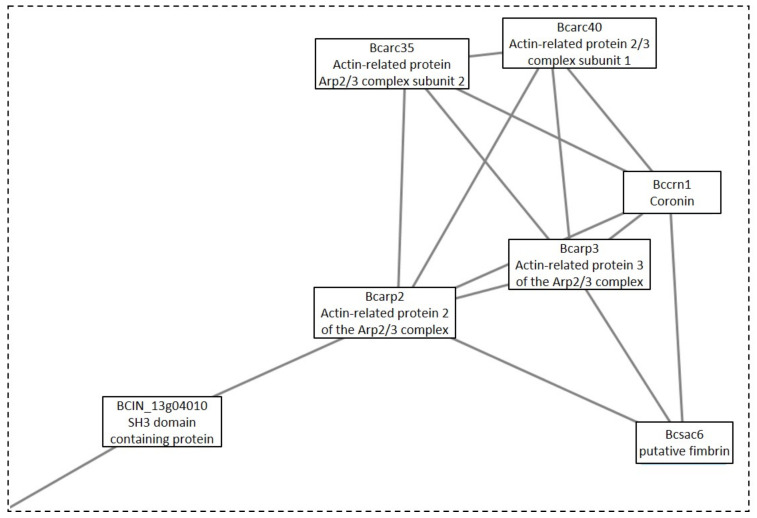
Section A from Cluster 10 (Figure 7).

**Figure 9:**
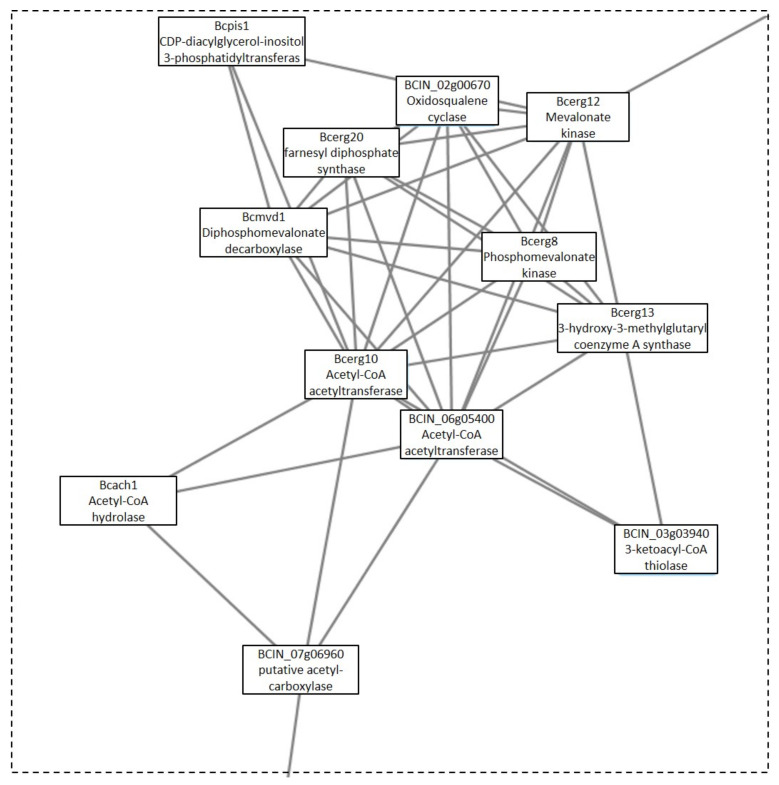
Section B from Cluster 10 (Figure 7).

**Figure 10:**
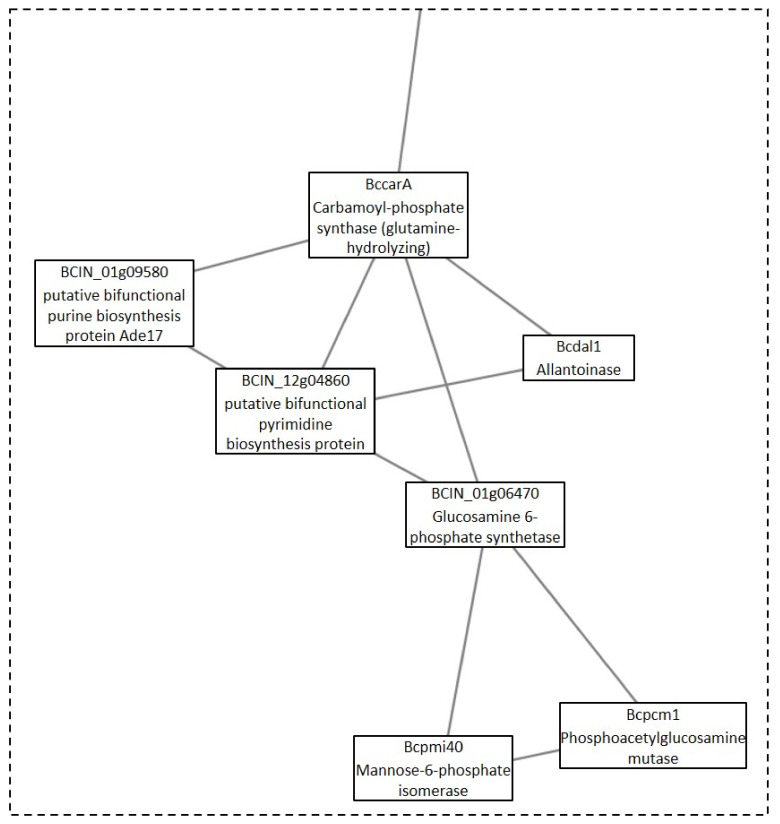
Section C from Cluster 10 (Figure 7).

One additional cluster under the GLU condition was analyzed, due to its possible implication in toxin production. This cluster was Cluster 10, which was composed of three linked sections (Figure 7). Each section was responsible for different biological processes. Section A (Figure 8) is made up of proteins that are expected to participate in actin polymerization, endocytosis, and cell wall integrity (Bcarp2, Bcarp3, Bcarc35, Bcarc40, Bccrn1, Bcsac6, and BCIN_13g04010) in comparison with yeast orthologs [21]. The link in this section, with section B (Figure 9), is BCIN_13g04010, which is a SH3 domain containing protein. Section B (Figure 9) includes six out of the seven enzymatic steps involved in the biosynthesis of isopentenyl pyrophosphate (IPP) via the mevalonate (MVA) pathway (Bcerg10, BCIN_06g05400, Bcerg13, Bcerg12, Bcerg8, Bcmvd1); and the enzymatic step to the biosynthesis of farnesyl pyrophosphate (FPP) (Bcerg20), the immediate precursor essential for the biosynthesis of terpenes in fungi, including *B. cinerea* [22]. Additionally, two genes encoding acetyl-CoA acetyltransferase (Bcerg10 and BCIN_06g05400) and a 3-ketoacyl-CoA thiolase were present in the cluster. Finally, two enzymes implicated in regulating the intracellular acetyl-CoA pool and fatty acid (BCIN_07g06960 and Bcach1) connect this section with the third section of the cluster (section C, Figure 10). Section C (Figure 10) from Cluster 10 have seven proteins related to amino acids and nucleotide metabolism, and to the synthesis of cell wall component precursors (BccarA, Bcdal1, BCIN_01g09580, BCIN_12g04860, BCIN_01g06470, Bcpmi40, and Bcpcm1).

#### 3.2.2. Cluster Analysis under TCW Condition

**Figure 11:**
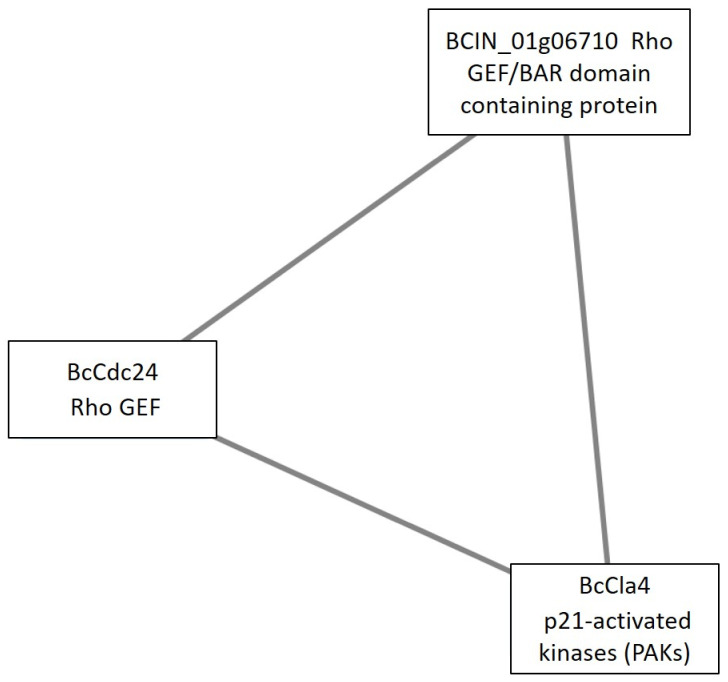
Cluster 14: signaling cluster under the TCW condition. Clusters were obtained analyzing STRING networks of proteins, exclusively identified or overexpressed in *B. cinerea* proteomes (membranome, phosphoproteome, phosphomembranome, and secretome) under TCW with MCODE.

Under the TCW condition, only one cluster related to signaling was observed, Cluster 14 (Figure 11). This cluster was composed of three proteins, all of them related to small GTPases cascades (Bccla4, Bccdc24, and BCIN_01g06710). The third component of the cluster was the hypothetical protein BCIN_01g06710. After a Blastp analysis of this protein, the most similar protein not annotated as a hypothetical protein in the non-redundant (nr) protein sequences database of NCBI, was a Rho guanyl nucleotide exchange factor protein from *Rutstroemia* sp. NJR-2017a BBW (accession number: PQE12603.1) (59.93% identity and 100% of query cover). Additionally, the NCBI Conserved Domain Search analysis tool detected the bin/amphiphysin/rvs (BAR) domain of the dynamin binding protein (cd07589: BAR_DNMBP) as the best hit. The second-best hit was a RhoGEF domain (cd00160), which is present in the guanine nucleotide exchange factor for Rho/Rac/Cdc42-like GTPases. 

## 4. Discussion

### 4.1. GO Analysis

GO analysis was performed using exclusive or overexpressed proteins under each condition (GLU and TCW) identified in several *B. cinerea* proteomes [6,9,12], analyzing them as a whole proteome. This analysis was performed to determine the relevance of *B. cinerea* proteins affected by the changes in the used carbon source, which represent two pathogenic states (GLU as a constitutive stage; and TCW as a virulence inductor). To this aim, proteins identified under each condition were categorized according to their specific gene ontology (GO) annotations, by MF and BP; and SEA analysis was performed. GO results showed a more active and variated metabolism when an easy assimilable carbon source was present in the culture medium, which agree with data obtained from the *B. cinerea* membranome and phosphoproteome, but not with the phosphomembranome, when they were independently analyzed [9,12]. Phosphomembranome was the only proteome that presented a higher number of GO categories under TCW conditions, which may indicate a crucial role as a switch in the change between pathogenic states of those membrane proteins regulated by phosphorylation. Thus, as well as inactivating the constitutive metabolism under the GLU condition, the fungus needs to activate others strategies to infect the host when TCW is present, such as the production of cell wall degrading enzymes [10]. However, the main proteome reaction is a less active metabolism under the TCW condition, which coincides with the last results describing the interactions between Botrytis and its host as subtle, elegantly manipulating crucial biological processes in host plants for its own success [23]. 

Under the GLU condition, proteins related to TOR signaling were identified: (i) a phosphatidylinositol 3-kinase TOR protein (BCIN_01g11360), which was previously reported in *B. cinerea* as the main component of the TOR complex (BcTOR) [24]; (ii) a hypothetical protein BCIN_02g06630 (putative Slm1), a putative effector of the TOR complex [25]; and (iii) Bcsec13, a potential activator of TOR signaling [19]. In yeast, Slm1 and Slm2 proteins act downstream of TORC2 kinase pathways to control actin cytoskeleton organization, endosome trafficking, and recycling [25]. On the other hand, yeast Sec13 is a component of SEACAT (Seh1-associated complex subcomplex activating TORC1), an upstream element of the yeast TOR pathway [19]. The presence of these proteins under the GLU condition must mean an activation of TOR signaling. The TOR signaling pathway is implicated in controlling cell growth, proliferation, transcription, translation, autophagy, and metabolism processes in many eukaryotes. In *B. cinerea*, inhibition of TOR generates reduction and even loss of infectivity and severe suppression of conidiation [24]. In the same way, TOR inhibition reduces pathogenicity in *F. graminearum* by regulating mycelial growth and virulence [26]. Along with the knowledge that TOR is activated by both nitrogen and carbon metabolites (such as glucose), and promotes energy-consuming processes (cell division, mRNA translation, and anabolism), it is possible that TOR could be crucial to botrytis toxin production, which is known to be active only under GLU conditions in *B. cinerea* [10].

In addition, GO annotation in GLU revealed proteins related to the phosphorelay signal transduction system category: Bchhk2 (HHK2, histidine kinase-group V protein); Bcrim15 (serine/threonine protein kinase RIM15); and Bcbos1 (histidine kinase). It has been described that *B. cinerea* contains two RRs (Bcrrg1 and Bcskn7), a single HPT, and at least 20 HK-encoding genes, which allow the integration of multiple input signals into a single response. In *B. cinerea*, the knowledge of HK functions is limited, and only three HK-encoding genes have been characterized (bchk1, bchk5, and bcos1). The deletion of bcos1 results in the constitutive activation of Bcsak1 and increases sensitivity to hyperosmotic stress, oxidation, decreases sensitivity to fungicides, and impairs virulence and loss of conidiation [27]. In this paper, two HKs (Bcbos1 and Bchhk2) and a putative RR (BcRim15) under GLU condition were identified. Neither Bchhk2 nor Bcrim15 were previously described in this fungus. Bchhk2 was previously classified as a group V fungal HHK, such as MoHik5p from *Magnaporthe oryzae*. MoHik5p was required for pathogenicity in *M. oryzae*, and its deletion produced reduced vegetative growth, lack of conidiation, cell wall instability, stress hyper-susceptibility, and loss of virulence in the fungus [28]. Group V fungal HHKs were described in filamentous Ascomycota, Basidiomycota, and in early diverging fungi, but their functions remain largely unclear, as only MoHik5p has been functionally characterized [17]. On the other hand, in the genomes of Saccharomycotina. Four RR proteins have been identified (Srr1, Skn7, Ssk1, and Rim15); and in *C. albicans*, the genes that encode putative RRs include RIM15 [29]. In yeast, Rim15 is a master regulator of different nutrient-sensing pathways, being phosphorylated and negatively-regulated by TOR (thought Sch9) and PKA under nutrient availability, which regulates hyphal growth [22,30]. In *B. cinerea*, Bcrim15 was found involved in oxalic acid (OA) biosynthesis regulation and pathogenesis thought interaction with Bcmkk1. Bcmkk1 is a component of the cell wall integrity (CWI) pathway, that interacts with and activates Bcrim15 by impeding the phosphorylation of Bcrim15 by Bcsch9; thus, negatively regulating OA biosynthesis [31]. However, the description of Bcrim15 as a RR component of the two-component phosphorelay system of *B. cinerea* was not reported. This, joined to the implication of Bcrim15 in the production of a secondary metabolite (OA), and its presence in the phosphoproteome of *B. cinerea* under the GLU condition (induction of botrydial and dihydrobotrydial biosynthesis condition) [8,12], may indicate a potential role in the regulation of *B. cinerea* toxins biosynthesis, as well as could be implicated Bchhk2.

The last of the three most relevant GO categories, exclusively presented under GLU conditions, is the inositol lipid-mediated signaling category (include phosphatidylinositol (PI)-mediated signaling), containing two proteins: (i) Bcstt4 (phosphatidylinositol 4-kinase STT4); and (ii) a putative phospholipase D1 (hypothetical protein BCIN_09g01240). PIs are important signaling molecules, having essential roles in the regulation of many biological processes, just as membrane transport. Phosphatidylinositol 4-kinases (PI4Ks) catalyze the first step in the biosynthesis of four of the seven PI lipids, generating phosphatidylinositol 4-phosphate (PI4P). In *F. graminearum*, FgLsb6, a PI4K, is involved in vegetative growth, pathogenicity, and toxin deoxynivalenol (DON) production, by regulating endocytosis through PI4P [32]. On the other hand, it was also reported that Stt4 is implicated in virulence in *C. albicans* [33]. Additionally, under GLU conditions, a putative PLD (BCIN_09g01240, XP_001547853.2) has been identified. Activated PLD enzyme hydrolyses membrane phospholipids (PC, PE, and PI) and releases phosphatidic acid (PA). PA itself acts as an intracellular messenger (actin-cytoskeleton reorganization, cell proliferation, DNA synthesis, and secretion) or is further metabolized into second messengers, DAG, and lysophosphatidic acid (LPA). Some examples of PLD isoforms affecting fungal pathogeny and virulence have been reported [34], including *F. graminearum* and *B. cinerea*. *B. cinerea* possess a PE specific PLD (BCIN_02g01720, XP_001558947.1), which regulates the expression of ACP1, which is a protease secreted during the *B. cinerea* infection process. In addition, the phytotoxin botrydial triggers PA production via plant cells, PLD and phospholipase C/diacylglycerol kinase activation in tomato cell suspensions [35]. In *F. graminearum,* FgPLD1 deletion resulted in decreased production of the mycotoxin DON, showing decreased virulence in infection of wheat. However, deletion of FgPLD2 and FgPLD3 showed no reduction of DON production and virulence, indicating differential roles of the PLD genes in the pathogenicity of *F. graminearum* [36]. Thus, the putative PLD identified in our analysis (BCIN_09g01240, XP_001547853.2) could be a new component in the signaling pathways involved in regulation of *B. cinerea* toxin production, as well as Bcstt4, while previously identified *B. cinerea* PLD (BCIN_02g01720, XP_001558947.1) regulates secretion of lytic enzymes. In this scenario, PLD and Bcstt4 may be acting in the same pathway or independently thought PA and PI4P as secondary messengers, respectively. 

There were nine other (less abundant) GO categories exclusively represented under GLU conditions. Seven of these categories are related to the regulation of apoptotic signaling. It is important to highlight that, under TCW, one category relating to apoptotic signaling was identified. This category is different from the apoptotic categories identified under GLU, and they are represented by different proteins: Bclhs1 under GLU; and Bcire1 under the TCW condition. Both proteins are related to UPR and apoptotic signaling. Bcire1 is known to be a core regulator of unfolded protein response (UPR) during ER stress in *B. cinerea* [37]. In yeast, it was previously reported that Ire1 is an activator of the UPR pathway that promotes the activation of the chaperone gene transcription, such as Lhs1, to relieve the ER stress, avoiding apoptosis [38]. However, in mammalian cells, a prolonged activation of Ire1 (phosphorylated Ire1) coulf trigger apoptosis in cells under certain physiologic and pathophysiologic conditions [39]. Compared to the large amount of knowledge of UPR in human and plant systems, UPR has only been slightly described in a small number of fungal pathogens, including *Aspergillus fumigatus*, *Alternaria brassicicola,* and *Ustilago maydis* [40,41], and all of them demonstrate that the regulation of UPR is associated with fungal pathogenicity [40,41]. In fact, both proteins were reported to play an important role in fungi virulence [40], but it was not described in botrytis. Thus, a functional UPR, and its correct regulation in *B. cinerea,* is crucial to the pathogenesis, as well as in other fungal pathogens, with BcLhs1 and BcIre1 highlighted as the key components.

Finally, under TCW as the sole carbon source, key components of the cAMP pathway, such as cAMP-dependent PKA, must be inactive due to the presence of the regulatory subunit BcPKAR, without identification of the catalytic subunit Bcpka1, which was quite the opposite from what happened under the GLU condition. In *B. cinerea*, Bcpka1 regulates growth, mainly, the late stages of the infection, without affecting botrydial biosynthesis [42]. This agrees with the activation of PKA under GLU conditions, where the fungus may understand that it is inside the plant cell, after degrading and penetrating the cell wall with the consequent release of simple sugars.

### 4.2. Protein Interaction Analysis

Exclusive or overexpressed proteins identified under each condition (GLU or TCW) in *B. cinerea* proteomes previously performed by our groups [6,7,9,12] were used to perform a protein interaction analysis using the STRING database; and a clustering analysis using Cytoscape [15,20]. The analysis of these proteomes as a whole was done in order to better understand the signal transduction cascades differentially expressed by the type of carbon source, identifying new protein–protein interactions that could not be identified in the individual analysis of each proteome. The STRING interaction analysis results showed more complex networks with more nodes and connecting edges for TCW (Supplementary Table S3) and GLU (Supplementary Table S4) than previous analyses of the integral proteomes. These networks went through a deeper analysis to identify clusters by using the software MCODE [16]. This analysis identified 14 and 41 clusters in TCW and GLU, respectively (Supplementary Tables S5 and S6), showing more cluster numbers in each condition than the individual analysis of each proteome on its own [7,9,12]. The number of clusters relating to signal transductions were higher too, with three clusters under the GLU condition and one under TCW. In addition, one cluster relating to toxin production was identified under the GLU condition. None of those clusters were previously detected in the individual analysis of each proteome, only a minimal portion of Cluster 15 [12]. Thus, this integrating proteome analysis provides a (possible) higher understanding of the signal transduction changes in *B. cinerea* during infection.

#### 4.2.1. Clusters under GLU Condition

The analysis of the first cluster related to signal transduction, Cluster 15, showed that it was composed of small GTPases signaling (Bcac, putative Cdc25 GEF), cAMP pathway (Bcpka1), MAPK cascades (Bcsak1, Bcos4), and two-component signal transduction system (Bcos1). This cluster showed interactions between Bcsak1, Bcos1, and Bcpka1 (cAMP-dependent protein kinase catalytic subunit). A link between Bcsak1 and the cAMP-dependent PKA pathway was previously detected in *B. cinerea*, where a proteomic analysis showed a negative regulation of the cAMP pathway, independently of Bcos1, by the MAPK Bcsak1. This result was reflected by higher intracellular cAMP levels and the overproduction of BcPKAR proteins in the Δ*bcsak1* mutant, but not in Δ*bcos1* [25]. In a similar way, the pathogenic fungus *Cryptococcus neoformans* showed antagonism between the PKA and Hog1 (called Bcsak1 in *B. cinerea*) controlling ribosome biogenesis via mRNA stability in response to glucose availability. Glucose signaling through PKA stabilized ribosomal protein (RP) mRNAs whereas glucose starvation (induced oxidative stress response genes) destabilized RP transcripts through Hog1 [43]. The cAMP/PKA pathway in *C. neoformans* plays a well-documented role in virulence [44], as well as in *B. cinerea* [45]. On the other hand, interaction of the histidine kinase (HK) Bcos1 and Bcsak1 was previously reported. Bcos1, which is devoted to the adaptation to osmotic and oxidative stress, is known to negatively regulate Bcsak1 by the inactivation of the cascade phosphorylation of the MAPK pathway (Bcos4, Bcos5 and Bcsak1) in the absence of osmotic or oxidative stress [27,45]. Additionally, although the Bcos1–Bcsak1 signaling pathway clearly regulates ionic and oxidative stress, macroconidia production, penetration capacity, and plant necrosis in *B. cinerea*, there are functions potentially controlled by Bcos1 in a Bcsak1-independent way. These results indicate that Bcsak1 is not the only downstream effector of the Bcos1 HK [27]. In particular, proteomics analysis using *B. cinerea* Δ*bcos1* and Δ*bcsak1* mutants revealed that Bcos1 and Bcsak1 have regulatory roles in botrydial biosynthesis, with the histidine kinase Bcos1 as a positive regulator and MAPK Bcsak1 a negatively regulator. Finally, it is important to highlight that Bcsak1 was detected in the phosphomembranome analysis under GLU and TCW conditions, but with a different phosphorylation pattern. One explanation for the differences in the pattern of phosphorylation was the presence of Bcos5 under TCW, but not under GLU conditions [9]. Finally, the presence of the MAPKKK Bcos4 in the cluster comes from its identification in the membranome of *B. cinerea*, so this protein must be in its unphosphorylated (inactive) form and someway linked to the membrane [7]. Thus, under the GLU condition, where it was reported that botrydial and dihydrobotrydial is synthetized [10], Bcos1 must be active and negatively regulating the phosphorylation of the Bcsak1 by MAPK pathway (Bcos4, Bcos5, and Bcsak1), activating botrydial and dihydrobotrydial biosynthesis. On the contrary, Bcpka1 may be phosphorylating Bcsak1 in a different way, giving this protein a new role in signal transduction regulation, depending on its phosphorylation state, which must be better studied. 

Another interaction presented in Cluster 15 is the connection between the small GTPase Bcrac and the MAP kinases (Bcsak1), agreeing with the results by Kilani, J. et al., where the Bcrac effector Bccla4 abundance decreased significantly in Δ*bcsak1* mutants [25]. In *B. cinerea*, Bcrac is implied in cell cycle control and growth [46]. Orthologs of both proteins in yeast, Rac and Sak1, are implicated in the control of the cell division in this organism [46]; therefore, the interaction between them is reasonable, but it was not previously described in *B. cinerea.* Bcrac showed a potential link with BCIN_08g04300, which is also connected to Bcpka1. Analysis of BCIN_08g04300 sequences showed that this protein belongs to the same subfamily of *Saccharomyces cerevisiae* guanine nucleotide exchange factors (GEFs) Sdc25 and Cdc25. Cdc25 in *S. cerevisiae* is hyperphosphorylated by Pka1 in response to glucose, which inhibits RasGEF activity and affinity for Ras, as well as serves as a negative feedback mechanism, by which the intracellular cAMP synthesis is inhibited by PKA through Cdc25p. In addition, the RasGEF FgCdc25 of *Fusarium graminearum* regulates its development and virulence via cAMP and MAPK signaling pathways [47]. Sak1 phosphorylation by Pka1, instead of by BcOs5 (Bos5), and the presence of BcRac, and a putative Cdc25 GEF, must indicate activation of cell division and growth under nutrient rich conditions. Less is known about the two last members of this cluster, glycogen synthase kinase 3 (GSK3) and PP1C. In yeast, a GSK-3 homolog is necessary for cytokinesis and chromosome segregation. Additionally, GSK-3 is the mammalian member of a highly conserved family of protein serine/threonine kinases implicated in glycogen synthesis, cell fate determination, nuclear signaling, and hormonal regulation [48]. The FGK3 glycogen synthase kinase gene orthologous to mammalian GSK3 of *Fusarium graminearum* was identified as an important virulence factor, being important for growth, conidiogenesis, DON production, pathogenicity, and stress response [49]. Finally, BCIN_06g02220 encoding a putative serine/threonine protein phosphatase PP1C could be implicated in the regulation of Bcsak1 and GSK3 by its dephosphorylation. In eukaryotes, PP1 is involved in the regulation of glycogen metabolism, muscle physiology, RNA processing, protein synthesis, transmission of nerve signals, induction of apoptosis, and control of cell cycle [50], making understandable its presence in this cluster, and its potential interaction with the putative GSK3 and Bcsak1. In addition, a PP1-related PPases, Ppz1 was demonstrated to be involved in cation homeostasis, cell wall integrity, and virulence in *Candida albicans* [51]. Hence, BCIN_08g04300 (putative Cdc25), BCIN_16g04330 (putative GSK3) and BCIN_06g02220 (putative PP1C) are proposed, for the first time, as virulence factors in *B. cinerea,* considering that they are expressed in the same conditions of botrydial and dihydrobotrydial biosynthesis, and were reported to be implicated in virulence in other pathogenic fungi.

The second cluster related to signal transduction under the GLU condition was the cluster 24. This cluster presented, as a central linker, a phosphatidylinositol 3-kinase tor2 (BCIN_01g11360), which was previously reported in *B. cinerea* as the main component of the TOR complex (BcTOR) [24]. Eukaryote functions of TOR and its role in regulating growth and the infection process in *B. cinerea* was previously presented and discussed in the GO analysis section. By its predicted interaction with putative SNXs, Atg18, Atg11, Dnm1, Kcs1, and Arg82, joined to the well-known fuction of these protein in yeast; *B. cinerea* phosphatidylinositol 3-kinase tor2 potentially regulates membrane traffic and protein sorting in the endosomal system [52]; protein recycling; vacuolar protein sorting [53]; cytoplasm to vacuole transport; starvation-induced autophagy [31,54]; and vacuolar morphogenesis [55]. Another conserved function of TOR is regulation of transcription [56], explaining the presence of the third group of proteins of this cluster (BCIN_07g05050, BCIN_02g06790, Bcrtf1, Bcctr9). In yeast, RNA polymerase-associated protein CTR9 and RNA polymerase-associated protein RTF1 are components of the multifunctional complex PAF1, which is involved in transcription, elongation, and transcription-coupled histone modification, such as ubiquitination of ‘Lys-126’ histone H2B [57]. In sum, this cluster highlights that tor2 phosphatidylinositol 3-kinase, as a component of both the TORC complex in *B. cinerea*, must regulate multiple cellular processes to control cell growth, such as inhibition of starvation-induced bulk autophagy under glucose availability, as with *Saccharomyces cerevisiae* [56]. These results indicate an active recycling of cellular components, which agree with an active metabolism in glucose controlled by the central regulator BcTOR complex. This role, as the central regulator of metabolism, agrees with BcTOR, as previously reported as an efficient target in the control of gray mold because of its implication in the infection process of *B. cinerea* [24]. Finally, Bckcs1, an inositol hexakisphosphate kinase 1 that transforms InsP6 to InsP7/PP-InsP5 is proposed as a key component in the regulation of *B. cinerea* virulence, since PP-IP5 (IP7) was described as the most crucial IP species for fungal fitness and virulence [58]. Taking into account their potential functions and their identifications under GLU conditions, BcTOR and Bckcs1 could also be implicated in botrydial and dihidrobotrydial synthesis regulation.

Cluster 36 was the last signaling cluster identified under the GLU condition and it is composed of a small GTPase cascade (Bclrg1, Bcrho1, and BCIN_05g06700) and mRNA decay components (Bcpat1, Bclsm1, and Bccdc39). In *S. cerevisiae,* Lsm1 is a component of the cytoplasmic LSM1–LSM7 complex, which is involved in mRNA degradation by activating the decapping step [59]. Pat1 is an activator of decapping in this yeast, interacting and recruiting the LSM1–LSM7 complex to P-bodies [60]. In *S. cerevisiae,* Cdc39 acts as component of the CCR4-NOT core complex, which is a general transcription factor (in the nucleus) and a mRNA deadenylase involved in mRNA turnover (in the cytoplasm). The NOT protein subcomplex negatively regulates the basal transcription of many genes [61]. Thus, in yeast, these proteins act as general mechanisms of translational and transcriptional repression. On the other hand, small GTPase cascade components identified in this cluster were related with the Rho1 signal. This included two Rho GAP, Bclrg1, and BCIN_05g06700. *S. cerevisiae*, *M. oryzae,* and *N. crassa* Lrg1 orthologs were functionally characterized. In *S. cerevisiae*, Lrg1 activates CDC42, RHO1, and RHO2 [62]. In *M. oryzae,* Lrg1 acts as a Rho GAP [63]. In *N. crassa*, Lrg1 acts as a Rho1-specific GAP [64]. Additionally, BCIN_05g06700 showed Rho1GAP and GEF domains, so it may deactivate or activate Rho1 [65,66]. Rho1 regulates the activity of the (1,3)-β-D-glucan synthase and the cell wall in *S. cerevisiae* [67]; polar tip growth in *N. crassa*; cell wall integrity, and stress response in *Aspergillus fumigatus* [68]; and morphogenesis and cell wall biosynthesis in *Fusarium oxysporum* [69], but has not been described in *B. cinerea*, neither the fuction of Lrg1. The interaction between these two pathways (Rho1 signal and mRNA decay) was predicted through Bccdc39 (component of the CCR4-NOT core complex in yeast) and the RhoGAP Bclrg1. It was reported that inactivation of gene encoding subunits of Ccr4-NOT in *C. albicans* and *C. neoformans* reduces their virulence [70]. This could be due to the connection of this complex with Rho1, reported in *S. cerevisiae* by Ito et al. (2011) [71]. These authors reported that, in *S. cerevisiae*, the CCR4-NOT complex modulates a signal from Rho1 in the CWI pathway, by regulating the expression of Rho1 GEF and GAP (LRG1) [71]. In botrytis, Bcrho1 was not described as a virulence/pathogenicity factor. However, it was described as a virulence factor in *Fusarium oxysporum* and *Aspergillus fumigatus,* without affecting the pathogeny [68,69]. This agrees with its identification in the botrytis proteome under the GLU condition, known as the condition of botrydial and dihydrobotrydial production in this fungus [10]. Additionally, in *M. oryzae,* Lrg1 (Rho1GAP) is involved in regulating vegetative growth, conidiation, appressorium formation, and pathogenicity [63]. These results highlight the importance in the progress of the infection process of the pathogenic fungi of Rho1 and its regulators. In this context, Bccdc39, Bclrg1, Bcrho1, and BCIN_05g06700 (Rho1GAP/GEF) are good candidates to be analyzed as pathogenic/virulence factors in *B. cinerea*.

Finally, Cluster 10 identified under the GLU condition was composed of proteins related to biosynthesis of toxin precursors (IPP and FPP) through the mevalonate pathway, which agrees with the previously reported induction of botrydial and dihydrobotrydial biosynthesis under this condition, and not under TCW [8]. Therefore, its deep analysis was performed in order to unravel new insights in botrydial and dihydrobotrydial biosynthesis regulation. One group of proteins identified in this cluster was hose related to actin polymerization, endocytosis, and cell wall integrity (Bcarp2, Bcarp3, Bcarc35, Bcarc40, Bccrn1, Bcsac6). In fungi, actin cytoskeleton is involved in cell polarity, cellular signaling, intracellular trafficking, cytokinesis, endocytosis, exocytosis, bud site selection, hyphal growth rates, polarity, cell wall remodeling, and cell shape determination [72]. Bcarp2, Bcarp3, Bcarc35, Bcarc40, and Bccrn1 are homologues of yeast Arp2/3 complex components [21]. In *Neurospora crassa,* the subapical patches of coronin (Crn1) colocalized with fimbrin, Arp2/3 complex, and actin, comprising the endocytic collar [73]. In addition, there are reports of fungal fimbrins, demonstrating that fimbrins are associated with endocytosis, hyphal growth, conidiation, cell wall integrity, secretion, and polarization, such as fimbrin Sac6 from *Candida albicans* [74] and FgFim from *Fusarium graminearum* [72]. In *Fusarium graminearum*, fimbrin (FgFim) is essential to toxin production. In this fungus, deletion of FgFim-15 indirectly affects DON biosynthesis, a sesquiterpene of *F. graminearum* reported as an important virulence factor [72]. The fimbrin Sac6 from the fungal pathogen *Candida albicans* was involved in regulation of secretion of lytic enzymes and virulence [74]. On the other hand, in the cluster, Bcsac6 is predicted to be linked to the mevalonate pathway by a putative Arp2/3 complex component (Bcarp2) and BCIN_13g04010 (SH3 domain containing protein). SH3 domains are protein interaction domains that play versatile and diverse roles in the cell, including the regulation of enzymes, changing the subcellular localization of signaling pathway components, and the formation of multiprotein complex [46]. The SH3 domain containing the protein of *Saccharomyces cerevisiae*, Ysc84p localizes to actin patches and plays an important role in actin polymerization during endocytosis and vesicle trafficking [75]. Furthermore, activation of actin polymerization by mammalian Arp 2/3complex is mediated by isoprenylated Rho proteins, which can be farnesylated or geranylgeranylation by geranylgeranyl or farnesyl transferases [76]. Interestingly, in *B. cinerea* proteomes, a Rho1 GTPase (Bcrho1) was identified under the GLU condition (cluster 36) as well as one putative farnesyl transferase (BCIN_05g03760, Supplementary Table S1B). Bcrho1 (M7UPQ4) is predicted to be connected to cluster 10 through Bcarp3 (M7UB19) and Bcarp2 (M7UBW4) (Supplementary Tables S4 and S6). Moreover, BcRho1 presented a predicted site of farnesylation using GPS lipid 1.0 software (high threshold). Thus, farnesylation of Bcrho1 under the GLU condition could be interconnecting toxin production by the mevalonate pathway with the actin polymerization function, such as secretion, indirectly regulating toxin production. In the same way, Bcsac6 and Bcarp2 may be part of this proposed regulation of toxin production, being proposed as potential virulence factors, just as with other fungal pathogens.

#### 4.2.2. Clusters under the TCW Condition

Under the TCW condition, only one cluster related to signal transduction was detected, which agrees with a less active metabolism under TCW, highlighted in GO results. This cluster was composed of three proteins related to small GTPases cascades: BcCla4; BcCdc24; and a putative fungal DNMBP (BCIN_01g06710). Cdc24 plays a central role in hyphal growth and establishment and maintenance of polarity in yeast and filamentous fungi, being a component of the polarity complex, just as Cla4 and other signaling proteins. As in most other fungi, BcCdc24 is part of a polarity complex and serves as a GEF for BcCdc42 and for Bcrac. Bcrac was reported to be essential to hyphal morphology, differentiation of conidia and sclerotia, and to infect. The most important differentiation process in pathogenic fungi based on polar growth is the development of functional penetration structures (requiring the reorientation of growth) and the colonization of the host tissue [77]. It was reported that the polarized growth of hyphae is slowed or completely arrested to generate different developmental structures, such as various types of conidiophores or infection structures [78]. In addition, phosphorylation of the exchange factor Cdc24 by the PAK-like kinase Cla4 may regulate polarized growth in yeast. Cla4 induces phosphorylation of Cdc24, leading to its dissociation from Bem1 at bud tips, thereby ending polarized bud growth [79]. On the other hand, BCIN_01g06710 was hypothesized to be a DNMBP, containing RhoGEF and BAR domains. BAR domain proteins are implicated in processes as fundamental and diverse, as fissions of synaptic vesicles, cell polarity, endocytosis, regulation of the actin cytoskeleton, transcriptional repression, signal transduction, etc. The BAR domain family of proteins are characterized by their modular architectures. In addition to the membrane-curvature sensing/inducing BAR domain module, most contain auxiliary domains, including SH3, PX, PH, RhoGEF, and RhoGAP domains. Human Tuba, which is a Cdc42-specific GEF, is the only BAR proteins with RhoGEF activity functionally characterized until now [80]. Tuba contains RhoGEF, BAR, and SH3 domains; however, this last domain is not present in our protein [81]. Interestingly, the other two Tuba-like proteins; Tuba3 and Tuba2, seem to be ubiquitously expressed in many cell-types of vertebrates. Both proteins lack the dynamin-binding region N-terminal SH3 domains, but very little is known about their biological functions [80]. Additionally, previous works showed that the fungal DNMBP family share similar functional domain organizations with human RhoGEFs, but it appears to lack the SH3 domain [82]. In *Talaromyces marneffei*, the GEF protein MsgA contains a DH domain, but a Bin-Amphiphysin134 Rvs (BAR) domain replaces the PH domain, unlike canonical GEFs. Deletion of *msga* results in aberrant yeast morphology during macrophage infection; and mutational analysis show that the BAR domain of MsgA is crucial in establishing correct yeast morphogenesis and localization during intracellular growth. Together, these results define a novel host infection specific pathway that regulates intracellular morphogenesis in *T. marneffei* [83]. Taking together all of this information, BcCla4, BcCdc24, and the putative fungal DNMBP (BCIN_01g06710) are proposed to be members of the *B. cinerea* polarity complex, with BCIN_01g06710 being a new component not previously reported on. Therefore, these proteins may be a crucial switch for changing polar growth to appressorium formation under the TCW condition. Lastly, only two of the three proteins in the cluster, BcCla4 and BcCdc24, were previously described as a pathogenicity factor in *B. cinerea* [46,77]. In this way, putative DNMBP is highlighted as a new potential pathogenicity/virulence factor.

## 5. Conclusions

The analysis of exclusive or overexpressed proteins under two carbon sources (GLU as a constitutive stage and TCW as a virulence inductor) identified in several *B. cinerea* proteomes, as a whole proteome, have provided new insight into the *B. cinerea* infection process, not shown in the individual analysis of each proteome. GO and cluster analyses showed some general conclusions: (i) the key proteomic reaction is a less active metabolism under TCW conditions, showing a smaller number of GO categories and a less complex network with a smaller number of clusters under this condition; and (ii) a possible role of phosphomembranome proteins as switches in pathogenic states, as this subproteome was the only proteome that presented a higher number of GO categories under TCW conditions. 

Deep analysis of the bioinformatics results allowed us to highlight new potential virulence/pathogenicity factors. The potential function as a virulence or/and pathogenicity factor was predicted based on the biological function of the identified protein in *B. cinerea* or/and its implication in other fungi virulence (such as toxin production) or pathogenicity. In addition, it is known that toxin production (botrydial and dihydrobotrydial) is active only under GLU conditions in *B. cinerea,* suggesting a connection between this culture condition and the identification of proteins implicated in toxin production (virulence factors). Following the said precepts, we have hypothesized new potential virulence factors that could regulate toxin production: (i) BcTOR; (ii) new potential components of the two-component phosphorelay system of *B. cinerea*, Bcrim15, and Bchhk2; (iii) components of the inositol lipid-mediated signaling, Bckcs1 (potentially regulated by BcTOR), Bcstt4, and a putative phospholipase D1 (BCIN_09g01240); (iv) BCIN_08g04300 (putative Cdc25); (v) BCIN_16g04330 (putative GSK3); (vi) BCIN_06g02220 (putative PP1C); and (vii) proteins implicated in cell wall integrity and secretion, Bcsac6, and Bcarp2. In this context, Bcpka1 is potentially regulating toxin production through phosphorylation of Bcsak1, BCIN_08g04300, and BCIN_16g04330 in response to glucose availability. Another interesting point in the toxin production research is the farnesylation of Bcrho1 under GLU conditions, which could interconnect and regulate toxin production with the actin polymerization function, implicating Bcsac6 and Bcarp2.

Other identified proteins could affect virulence or/and pathogenicity, such as: (i) BcLhs1 and BcIre1, which were highlighted as key components of UPR in *B. cinerea;* (ii) Bccdc39 (iii) Bclrg1; (iv) Bcrho1; and (v) BCIN_05g06700 (Rho1GAP/GEF).

Finally, the putative fungal DNMBP (BCIN_01g06710) was revealed as a novel pathogenicity of factors in *B. cinerea,* due to its potential implication in the *B. cinerea* polarity complex as a novel member, together with known members (BcCla4 and BcCdc24). It was reported that the regulation of the polarized growth of hyphae is essential to generate various types of infection structures. Therefore, BCIN_01g06710, BcCla4, and BcCdc24 may be crucial switches in pathogeny, changing polar growth to appressorium formation under TCW conditions.

## Data Availability

The original contributions presented in the study are publicly available. These data could be found here: PRIDE repository, (https://www.ebi.ac.uk/pride/archive/), with the dataset identifiers PXD003099 (membranome); PXD003099 (phosphoproteome); and PXD010961 (phosphomembranome). Secretome protein identification list is available in (Fernandez-Acero et al. (2010) [19]).

## Supplementary Materials

The following are available online at https://www.mdpi.com/article/10.3390/microorganisms9091837/s1, Figure S1: Supplementary Figure S1. Reduced Single Enrichment Analysis (reduced SEA) of GO Biological Process, Figure S2: Reduced Single Enrichment Analysis (reduced SEA) of GO Molecular Function, Table S1A. Exclusive or overexpressed proteins identified under TCW conditions in *B. cinerea* proteomes (membranome, phosphoproteome, phosphomembranome and secretome), Table S1B. Exclusive or overexpressed proteins identified under GLU conditions in *B. cinerea* proteomes (membranome, phosphoproteome, phosphomembranome and secretome), Table S2A: Single Enrichment Analysis (SEA), Table S2B: Reduced Single Enrichment Analysis (SEA), Table S3. String interactions results of proteins identifiend under TCW in *B. cinerea* proteomes (membranome, phosphoproteome, phosphomembranome and secretome), Table S4: String interactions results of proteins identifiend under GLU in *B. cinerea* proteomes (membranome, phosphoproteome, phosphomembranome and secretome), Table S5. Clustering analysis results using MCODE and String interactions network of proteins identifiend under TCW in *B. cinerea* proteomes (membranome, phosphoproteome, phosphomembranome and secretome), Table S6: Clustering analysis results using MCODE and String interactions network of proteins identifiend under GLU in *B. cinerea* proteomes (membranome, phosphoproteome, phosphomembranome and secretome).
