# Supplementary figures and images for: Deciphering the Dynamics of Signaling Cascades and Virulence Factors of B. cinerea during Tomato Cell Wall Degradation
Source: Microorganisms. 2021 Aug 30;9(9):1837. doi: 10.3390/microorganisms9091837 (PMC8466851; doi:10.3390/microorganisms9091837)

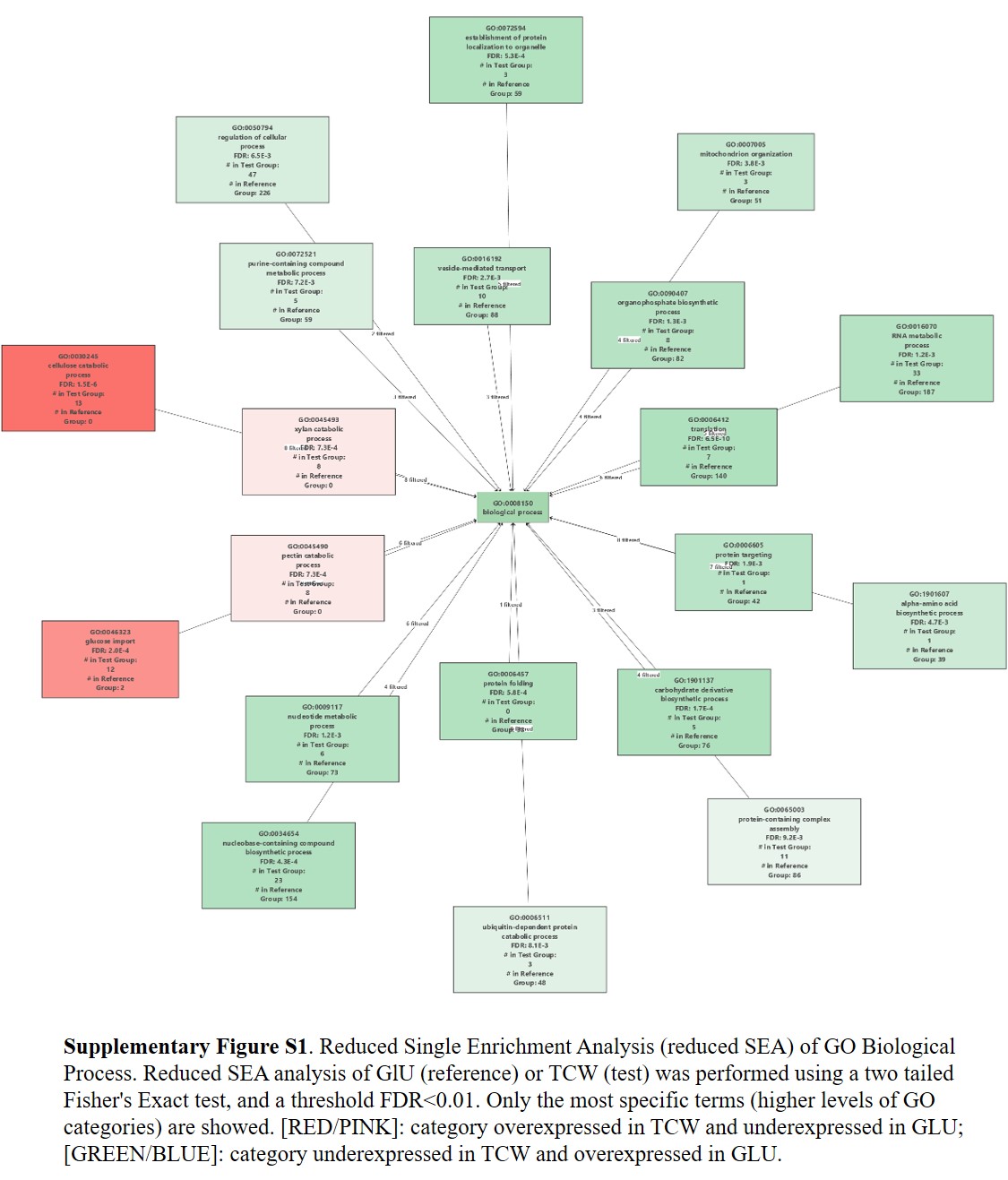

Supplement: Supplementary file 1 [file microorganisms-09-01837-s001.zip › Suplementary Figure 1.jpg]

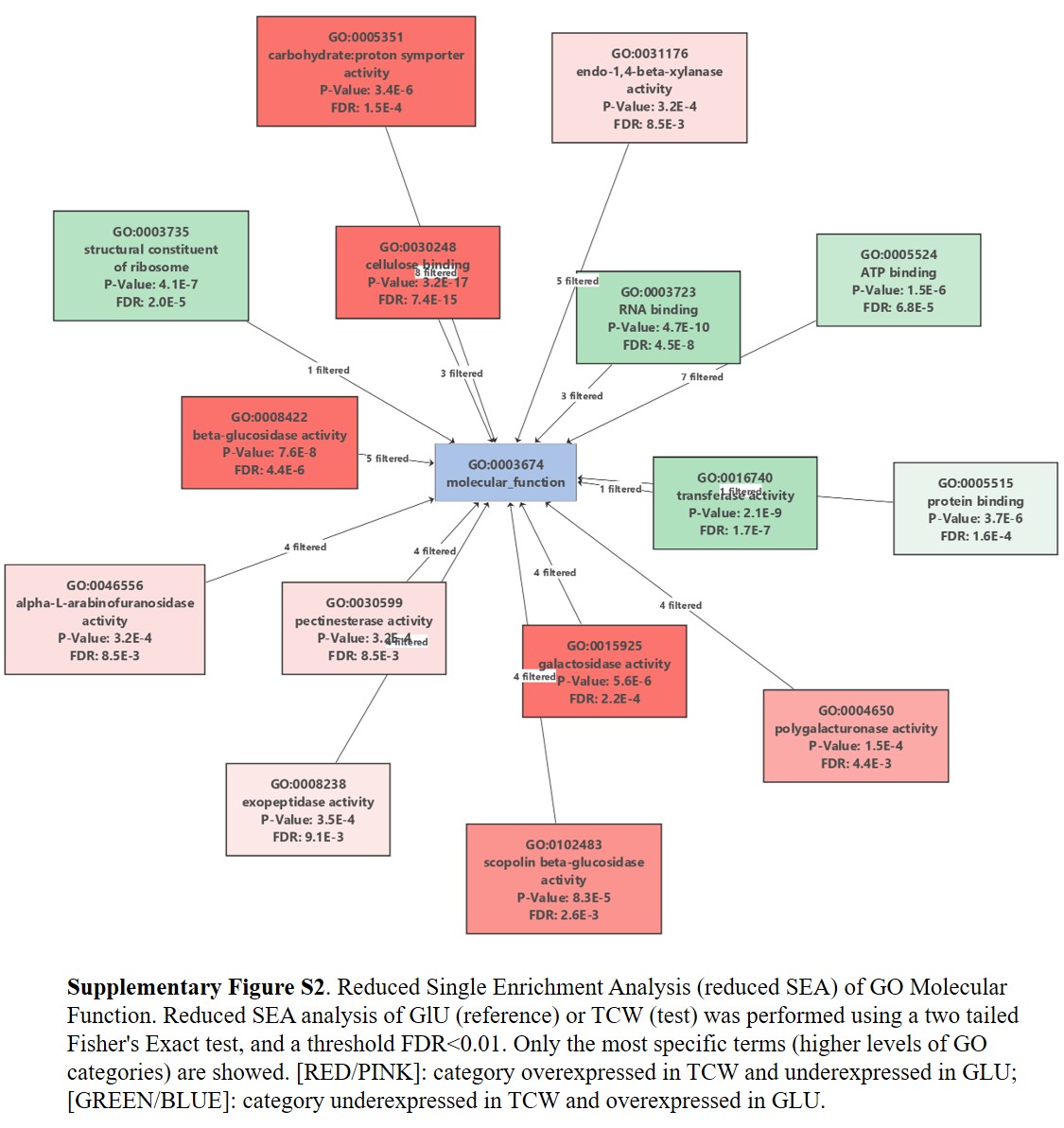

Supplement: Supplementary file 1 [file microorganisms-09-01837-s001.zip › Supplementary Figure 2.jpg]
